# Progress in Surgery of the Brain and Nerves

**Published:** 1902-09-27

**Authors:** 


*(To be continued.)*

### Progress in Surgery of the Brain and Nerves.

**Removal of Gasserian Ganglion.**—This operation is generally performed by removal of a considerable area of skull over the temporo-sphenoidal lobe, *i.e.*, the Hartley-Krause operation. Rose[1] advocates a modification of Doyen's method—that is, approaching it from below. He makes a sickle-shaped incision in the temporal region, commencing just in front of the lobule of the ear, and extending upwards to a point a quarter of an inch above the posterior extremity of the zygoma. Thence it is carried forwards parallel to the zygoma as far as the posterior extremity of the malar bone, and then upwards and backwards in a semi-circular direction to correspond to the temporal ridge on the frontal and temporal bones, ending at a point about an inch and a quarter above the upper extremity of the first vertical cut. The zygoma is divided at each end and together with the masseter is turned down. The coronoid process, together with the whole of the temporal muscle, is removed, and the external pterygoid is cleared from the great wing of the sphenoid. The trephine is applied just above the pterygoid ridge, at a point above the middle of the zygoma. The opening in the skull is then enlarged downwards towards the base until the foramen ovale is opened. The third division then comes into view. He claims that this method is much superior to the Hartley-Krause method, as not necessitating such an extensive removal of skull, and removing it from a less exposed position, he considers that it gives a much freer access to the ganglion and affords greater facilities for dealing with troublesome hæmorrhage. J. Hutchinson, jun.,[2] reports the results of five cases which after intervals of four, three, and two years are now quite cured. He strongly urges that the operation by the temporal route should alone be employed, and instances a case in which after an operation by the pterygoid route the neuralgia returned. A further operation was performed, the skull being opened by the temporal route and the Gasserian ganglion was found intact. He considers the removal of the ganglion with the second and third divisions sufficient, and advises that the first division be left. He relates a case in which the eye had to be removed after the first division had been divided. T. H. Morse[3] records two successful cases, one two years afterwards being quite free from pain. C. A. Ballance[4] recommends conducting the operation in two stages, with an interval of four or five days. Krause[5] records his experience based on 27 cases. He recommends the completion of the operation at one sitting. So far as the cure of the neuralgia is concerned, he has not had a single relapse. Three of his cases were fatal: one from collapse, one six days afterwards from a heart lesion, the third died three weeks afterwards from unknown causes. He considers that after the first few weeks eye complications need not be feared. Hæmorrhage during the operation is often troublesome; in some cases it has necessitated packing the wound and postponing the operation. Plugging the foramen spinosum is generally effectual in stopping meningeal hæmorrhage. Rose strongly urges that the operation is to be kept as a last resort. In his experience excision of the peripheral parts of the nerve is in many cases sufficient, and this should, he thinks, be always tried first. The lowest estimate of mortality is put at 10 per cent.; most writers put it higher.

**Cerebral Tumours.**—Among the achievements of cerebral surgery in recent years successful operations for cerebral tumours do not figure very highly; this is due not so much to difficulty in localisation as to difficulty in removal. Starr has analysed 1,100 cases and finds that only about 7 per cent. were "probably removable." Many tumours occur in parts of the brain in which they give rise to definite symptoms rendering localisation comparatively easy. Others occur in the so-called "silent regions" where localisation is not so easy. Among these silent regions is generally placed the prefrontal lobe. Dr. W. Elder[6] points out that this is fallacious; it is classified as silent because experiments have failed to elicit any definite motor reaction when this part of the brain has been electrically stimulated in monkeys. In man, however, tumours in this region do give rise to a definite group of symptoms characterised by loss of higher inhibitory control, causing loss of memory, attention, and power of judgment, together with the abolition of the sense of decency. He records a case under his care; the patient, a man æt. 47, had attacks of pain in the head, back, and legs, finally becoming localised in the left frontal region. He had occasional attacks of vomiting, but remained at work for six months when the symptoms became worse. He lost the sense of shame or modesty. Memory failed, and he was unable to contrast or compare, and therefore to form a proper judgment. Latterly, symptoms pointing to slight involvement of the motor fibres from the face and tongue centres, as shown by some slurring of the speech, but no aphasia; he had slight blurring of the right disc; finally he became comatose. Mr. Miles operated and removed a syphilitic growth from the left prefrontal region. The patient made steady progress after this and remained quite well. Mr. Miles[7] records a case of subcortical glioma removed from the left ascending frontal convolution. The patient a man, æt. 37, without previous warning, suddenly became speechless for about 20 minutes. There was a recurrence of the attacks every few days for a period of several weeks. Then followed headache, optic neuritis, and paresis of the lower facial muscles on the right side and weakness of the right half of the tongue. The "mutism" gave place to dysarthria, and finally almost complete anarthria. Two months after the operation the patient was practically well, only a little slowness of speech remaining. Dysarthria, he points out, is characteristic of lesions in this situation, not aphasia.

[1] Practitioner, May. [2] Lancet, November 30. [3] Ibid. [4] Ibid. [5] Edin. Med. Jour., September. [6] Brit. Med. Jour., February 1. [7] Edin. Med. Jour., April.

